# Meningitis and subdural empyema as complication of pterygomandibular space abscess upon tooth extraction 

**DOI:** 10.4317/jced.52916

**Published:** 2016-10-01

**Authors:** Paolo Cariati, Almudena Cabello-Serrano, Fernando Monsalve-Iglesias, Maria Roman-Ramos, Blas Garcia-Medina

**Affiliations:** 1 Oral and Maxillofacial surgery resident. Hospital Universitario Virgen de las nieves, Granada, Spain; 2 Maxillofacial Surgeon. Hospital Universitario Virgen de las nieves, Granada, Spain

## Abstract

Complication of dental infections might be various and heterogeneous. The most common complications are represented by maxilar celulitis, canine space celulitis, infratemporal space celulitis, temporal celulitis and bacteremia. Among rarest complications we found: sepsis, bacterial endocarditis, mediastinitis, intracranial complications, osteomyelitis, etc. Although dental infections are often considered trivial entities, sometimes they can reach an impressive gravity. In this regard, the present study describes a case of dental infection complicated by meningitis, subdural empiema and cerebral vasculitis. Furthermore, we observed other neurological complications, like thalamic ischemic infarction, during the disease evolution. Noteworthy, these entities were not presented when the patient was admitted to hospital. Therefore, the main aim of this report is to highlight the serious consequences that an infection of dental origin could cause.

** Key words:**Meningitis, subdural empyema, odontogenic infections.

## Introduction

Odontogenic infections rarely spread intracranially to cause complications such as thrombosis of the cavernous sinus, abscess, or meningitis. The incidence of morbidity and mortality is high because the diagnosis is often unsuspected. The literature reports a case of chronic meningitis in a patient with multiple oral infectious foci (1). In this case, samples of cerebrospinal fluid antigens against *S. milleri* were isolated. Then, remission of neurological symptoms was achieved through the eradication of oral infections (1). In the same line, literature also shows a case of cerebral abscess, of odontogenic origin, in a 46 years old man (2). Usually, the access road to the central nervous system is through a pansinusitis. In this case, a possible explanation of the infection diffusion is the meninges proximity. Moreover, a floor of the mouth celulitis might cause the diffusion of a dental infection to central nervous system. In fact, if the infection reaches the angular vein it could unchain a thrombosis of cavernous sinus (3). In the present work, we would like to stress that none of these mechanisms have been found. Many authors described intracranial and neurological complications caused by dental infections. The current treatment of this complication is based on the drainage or aspiration of the contents and antibiotics.

## Case Report

**Figure 1:**
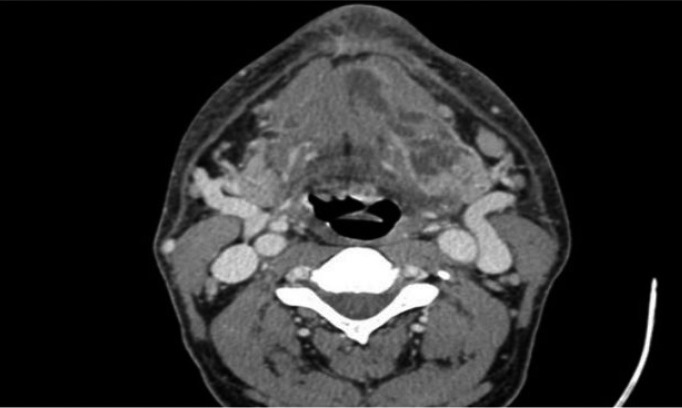
Floor of the mouth and pterygomandibular space abscess.

**Figure 2:**
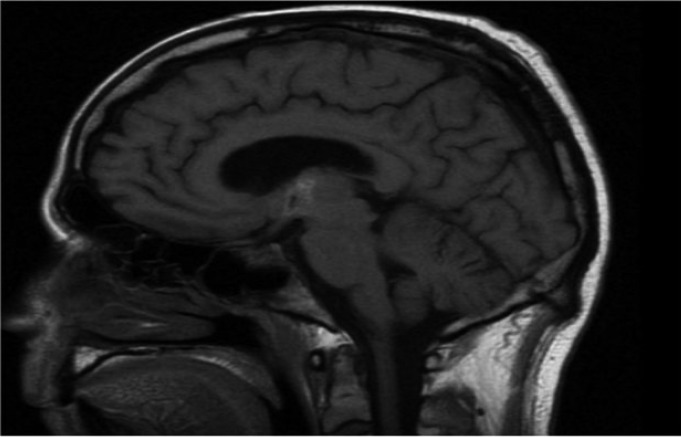
Temporal subdural empiema (left size).

**Figure 3:**
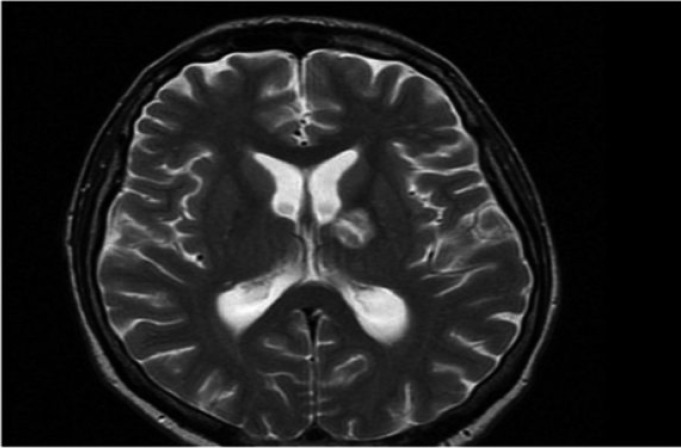
Temporal subdural empiema (left size).

The article describes a case of bacterial meningitis and subdural empiema upon surgical tooth extraction, in a 46 years old man. The patient arrived at the emergency service of the Hospital with temporomandibular pain, swelling of left temporomandibular zone and fever. We would like to emphasize that the only important information that the patient referred in the anamnesis was a tooth extraction two weeks ago (tooth 38). Taking in account these historical precedents, intravenous antibiotic treatment was administered and a CT of neck and facial area was carried out too. The analysis of the CT showed the presence of an abscess in the corner of the jawbone (left size). The patient was admitted in maxillofacial unit where the surgical drainage of the infectious collection was carried out. From this moment we decided to treat the patient with washed of the infected area every 8 hours and intravenous antibiotic treatment. Consequently, after 7 days the patient was discharged from the hospital with disappearance of the symptomatology and normalizing of analytical parameters. Convinced of our well performance, we were surprised to see the patient consulting again to the emergency service two weeks later. He came to the Hospital presenting identical symptoms of the previous time. In addition, his family referred the appearance of fluctuations in the level of consciousness. The exploration of the patient evidenced welling of the temporomandibular space and fever. Moreover, we noted the presence of a fistula with pus output in the preauricular zone (left size). A new CT reported signs of meninges inflammation. To examine it, we consulted to the neurological service of the Hospital. In this regard, Neurology unit decided to perform a lumbar puncture and bacterial meningitis of dental origin was diagnosed. Cocos gram + were found in the cerebrospinal liquor. In addition, a brain MRI revealed the presence of a temporal subdural empyema (left size). We notified the CT and MRI results to Neurology and Neurosurgery units that decided to prescribe a triple intravenous antibiotics treatment and surgical washed of temporomandibular space. However, despite these treatments the patient persisted with symptoms and had no improvement. Furthermore, he started to show behavioral disturbances alternating with fluctuations in the level of consciousness. Importantly, as complication of meningitis the patient developed a cerebral vasculitis and a thalamic ischemic infarction was evidenced by new MRI. Due to the the poor clinical evolution we consulted to the Infectious diseases unit. They reviewed the treatment and administered antifungal coverage. Noteworthy, the patient started to improve with the new treatment some days later. In fact, a serial cranial CT and brain MRI mirrored size reduction of empyema and cerebral vasculitis. After two weeks, the pus output through the skin of the temporomandibular area ceased and serials of Cranial CT and Brain MRI certified the resolution of the neurological complications. Lastly, the patient was discharged from hospital with a check-up appointment in 3 weeks to monitor the clinical evolution. It is important to highlight that during this check-up, we observed that the patient remained asymptomatic. Finally, we also would like to put the emphasis on the importance of the collaboration between several specialists in order to resolve these cases, (Figs. 1-3).

## Discussion

As we know, odontogenic infections are considered minor entities. However, they could be serious diseases. For instance, the literature describes many cases of dental infections complicated with serious pathologies. Haggerty CJ, DDS, MD,* and Tender GC, MD† J showed a case of subdural empyema of odontogenic origin caused by Actinomycotic infection (4). Santana-Cabrera L, Rodríguez-Escot C, Eugenio-Robaina P and Sánchez-Palacios M reported a case of Orbital cellulitis and subdural empyema as a complication of tooth extraction. According to the authors, the dissemination of the infection was through the blood circulation (5). Lee T *et al.* (6) reported a case of central condylar displacement with brain abscess from chronic mandibular osteomyelitis. In this study, the authors hypothesize that the patient’s chronic mandibular osteomyelitis led to glenoid fossa erosion with penetration of the germs in the middle cranial fossa and temporal lobe abscess formation.

According to the literature, pansinusit or thrombosis of cavernous sinus are usually the first steps for germs to reach the CNS. However, in our case, two Brain MRI and multiple Tc discarded that patient presented pansinusit, thrombosis of cavernous sinus or Condylar Displacement. According to us, the infection invaded the CNS by contiguity through the pterigomaxilar space and from there to the middle cranial fossa or across blood circulation. In addition, we suppose that other neurological complications developed by patient were a result of meningitis. In fact, Perry JR, MD; Bilbao JM, MD; and Gray T, MD published a case of bacterial meningitis complicated with cerebral vasculitis (7). The resolution of the case required a multidisciplinary approach. The equipment of neurology, neurosurgery and infectious diseases collaborated to resolve the case. In this circumstance the frequent surgical washing, antibiotic treatment guided by germs susceptibility and the cooperation among the various medical services of our Hospital were essential to eradicate the infection. The aim of this report was to eventide how dangerous could be an infection of dental origin. Infection, cellulitis and abscess of dental origin require aggressive antibiotic or surgical treatment according to the requirement of the case. This entity should never be underestimated. The proximity of the teeth to the cranial base explains how critical could become an untreated tooth infection. In conclusion, we invite all medical professionals to refer patients with dental infections with signs of severity to the emergency service. We should bear in mind that the presence of dysphagia, dysfonia, voice disorders, swelling face, fluctuations of mental status and high fever indicate the need for more aggressive treatment of infection.
